# Comparative Analysis of Brain Stiffness Among Amniotes Using Glyoxal Fixation and Atomic Force Microscopy

**DOI:** 10.3389/fcell.2020.574619

**Published:** 2020-09-11

**Authors:** Misato Iwashita, Tadashi Nomura, Taeko Suetsugu, Fumio Matsuzaki, Satoshi Kojima, Yoichi Kosodo

**Affiliations:** ^1^Korea Brain Research Institute, Daegu, South Korea; ^2^Developmental Neurobiology, Kyoto Prefectural University of Medicine, Kyoto, Japan; ^3^RIKEN Center for Biosystems Dynamics Research, Kobe, Japan

**Keywords:** mechanical property, brain morphology, force spectrometry, tissue mechanics, glyoxal fixation

## Abstract

Brain structures are diverse among species despite the essential molecular machinery of neurogenesis being common. Recent studies have indicated that differences in the mechanical properties of tissue may result in the dynamic deformation of brain structure, such as folding. However, little is known about the correlation between mechanical properties and species-specific brain structures. To address this point, a comparative analysis of mechanical properties using several animals is required. For a systematic measurement of the brain stiffness of remotely maintained animals, we developed a novel strategy of tissue-stiffness measurement using glyoxal as a fixative combined with atomic force microscopy. A comparison of embryonic and juvenile mouse and songbird brain tissue revealed that glyoxal fixation can maintain brain structure as well as paraformaldehyde (PFA) fixation. Notably, brain tissue fixed by glyoxal remained much softer than PFA-fixed brains, and it can maintain the relative stiffness profiles of various brain regions. Based on this method, we found that the homologous brain regions between mice and songbirds exhibited different stiffness patterns. We also measured brain stiffness in other amniotes (chick, turtle, and ferret) following glyoxal fixation. We found stage-dependent and species-specific stiffness in pallia among amniotes. The embryonic chick and matured turtle pallia showed gradually increasing stiffness along the apico-basal tissue axis, the lowest region at the most apical region, while the ferret pallium exhibited a catenary pattern, that is, higher in the ventricular zone, the inner subventricular zone, and the cortical plate and the lowest in the outer subventricular zone. These results indicate that species-specific microenvironments with distinct mechanical properties emerging during development might contribute to the formation of brain structures with unique morphology.

## Introduction

Although the vast majority of molecular machinery to generate neurons from progenitors are commonly conserved in amniotes (Englund et al., 2005; Martínez-Cerdeño et al., 2016; Nomura et al., 2016; Turrero García et al., 2016; Yamashita et al., 2018), the alignment of neurons in matured brains exhibits remarkable diversity (Medina and Abellán, 2009; Jarvis et al., 2013; Puelles et al., 2017; Cárdenas and Borrell, 2019; Pessoa et al., 2019). For instance, the mammalian brain has a six-layered structure, while the avian brains consist of compartmentalized nuclear slabs. During brain formation, newly generated neurons in the proliferative region [the ventricular zone (VZ) and subventricular zone (SVZ)] migrate to their final destinations. The mammalian neocortex (NCx) is originated in the most dorsal part of embryonic telencephalon (Puelles, 2013; Nieuwenhuys, 2017). In the mammalian telencephalon, most glutamatergic projection neurons are born in the dorsal proliferative region and migrate into the cortical plate (CP) radially (Nadarajah and Parnavelas, 2002; Noctor et al., 2004; Tabata et al., 2009), whereas GABAergic interneurons are born in the ventral proliferative region and migrate into the CP tangentially, resulting in a highly organized six-layered structure (Anderson et al., 1997; Batista-Brito and Fishell, 2009). Migrating neurons respond not only to biochemical signals but also to mechanical cues from distinct extracellular environments on the way to their destinations (Park et al., 2002; Huang, 2009; Honda et al., 2011; Long and Huttner, 2019). Indeed, intensive research using atomic force microscopy (AFM) has revealed the spatiotemporal diversity and crucial roles of the mechanical properties of the extracellular environment, especially stiffness, in the developing central nervous system (Elkin et al., 2007, 2010; Christ et al., 2010; Iwashita et al., 2014; Nagasaka et al., 2016; Thompson et al., 2019; Kjell et al., 2020). However, it remains unclear how stiffness controls cellular behavior to form species-specific brain structures.

To understand the role of stiffness in organizing diverse brain structures, a comparative analysis of stiffness in several animal brains is required. In this study, we examined the stiffness of pallia among amniotes: mice, turtles, songbirds, chicks, and ferrets. In general, living tissue should be used for stiffness measurements to obtain physiological profiles close to *in vivo*. However, there are practical difficulties in handling several kinds of living animals, such as breeding and shipping. Furthermore, stiffness measurements should be performed under identical experimental conditions and an identical AFM system to minimize deviations. Therefore, we examined whether fixed tissues could substitute for living tissues for stiffness measurements. For fixatives, we chose 4% paraformaldehyde (PFA), a common fixative, and 3% glyoxal, a novel fixative. Recent studies have demonstrated the powerful ability of glyoxal to preserve tissue and cellular structures (Bussolati et al., 2017; Richter et al., 2018).

Here, we confirmed that the macroscopic structure of a glyoxal-fixed brain was maintained as well as a PFA-fixed one. Surprisingly, our AFM measurements revealed that glyoxal-fixed brains showed much lower stiffness than PFA, conserving stiffness profiles similar to living brains, indicating that glyoxal fixation could be applicable to studying tissues’ mechanical properties. Based on this method, we found diverse stiffness patterns among amniote brains. The distinct mechanical properties of tissue microenvironments might provide different cues and scaffolds for neural cells and regulate their migrations to form diverse brain structures during development.

## Materials and Methods

### Animals

Animal protocols for mice and songbirds, including breeding and experiments, were approved by and performed according to guidelines of the Committee of Korea Brain Research Institute (KBRI). Pregnant ICR mice were purchased from Core Tech and bred in KBRI. The noon on which the virginal plug was detected was defined as embryonic day 0.5 (E0.5). The day of birth was defined as postnatal day 0 (P0). E16.5 embryos and 4 weeks juvenile mice were used in this study. Songbirds (*Taeniopygia guttata*) were raised in KBRI. Juvenile birds (30–40 days post hatch, dph) were used in this study. Other brains [turtle (*Pelodiscus sinensis*), chick (*Gallus gallus*), and ferret (*Mustela putorius furo*)] were obtained according to the guidelines of each institute (turtles and chicks, Kyoto Prefectural University of Medicine; ferrets, RIKEN, Center for Biosystems Dynamics Research).

### Preparation of PFA and Glyoxal Fixative

Paraformaldehyde (Merck, #8.18715) was dissolved in PBS (pH 7.4) at a final concentration of 4%. The glyoxal fixative solution was prepared according to published protocol (Richter et al., 2018). Briefly, 28 ml of ddH_2_O, 7.89 ml absolute of ethanol (analysis grade), 3.13 ml of glyoxal (Sigma-Aldrich, #128465), and 0.3 ml of acetic acid (Sigma-Aldrich, #A6283) were mixed well by vortex. After adjusting to pH 4.0 with 1 N of NaOH, the solution was filled up to 40 ml with ddH_2_O. The final concentration of glyoxal was 3%. Both fixative solutions were prepared on the day of the experiment and kept cool until use.

### Preparation of Fixed Brain Slices

Immersion fixation was applied in the embryonic stage for the mice and chicks, at 4 months and 2.5 years for the turtles, and at E35 and P0 for ferrets. Mouse embryos were taken from uteruses following the cervical dislocation of the mothers and kept in ice-cold PBS. Chick embryos were taken from fertilized eggs and fixed at 7 and 10 days of incubation at 37°C (E7 and E10, respectively) (Nomura et al., 2013). These stages correspond to the Hamburger and Hamilton stages (HH31-33 and HH36, respectively) (Hamburger and Hamilton, 1951). A ferret embryo was taken from the uterus, as previously described (Tsunekawa et al., 2016). The brains were dissected in ice-cold PBS and then transferred to the ice-cold fixative solution immediately. The turtles and one ferret at P0 were deeply anesthetized with isoflurane, and their brains were taken out. The brains in the fixative solution were put on a rotator in a cold room (4°C) overnight and then kept in PBS at 4°C until sectioning. The brains were cut into 300-μm-thick coronal sections in ice-cold PBS using a vibratome (Leica, VT1200S).

The transcardial perfusion was applied to juvenile animals. ICR mice were deeply anesthetized with intraperitoneal injections of pentobarbital (Entobar, HanLim Kharm, Co. Ltd., South Korea) and then perfused transcardially with either 4% PFA or 3% glyoxal (pH 4.0) fixative followed by PBS. The songbirds were deeply anesthetized with isoflurane (Hana Pharm Co. Ltd., South Korea) and then perfused like the mice. The brains were dissected out and post-fixed with fixative with the same perfusion overnight at 4°C and then kept in PBS at 4°C until vibratome sectioning.

### Preparation of Acute Brain Slices

All procedures were performed in ice-cold media according to a previous publication with slight modifications (Iwashita et al., 2014). Briefly, embryonic brains were dissected out in ice-cold DMEM/F12 (Sigma-Aldrich) containing D (+)-glucose and then embedded in 2% agar (Nacalai) in PBS. Embedded brains were cut into 300-μm-thick coronal sections in DMEM/F12/D (+)-glucose using a vibratome. Sections with agar frames were placed on a plastic dish coated with BD Cell Tak (BD Bioscience) and kept on ice until measurement. Before measurement, the dish containing the slices and media was allowed to reach room temperature.

To obtain the acute slices of juvenile mouse and songbird brains, artificial cerebrospinal fluid containing sucrose (slicing ACSF) was used to dissect and make vibratome sections. Brains were immediately dissected into ice-cold slicing ACSF and glued directly on the stage with cyanoacrylate glue. The brains were cut into 300-μm-thick slices in ice-cold slicing ACSF using a vibratome. Acute slices were incubated in slicing ACSF for 45 min and then transferred to measurement ACSF. The ACSF composition was described in a previous study (Kojima and Aoki, 2003). Supplementary Tables 1, 2 describe the media components.

### Measurement of Stiffness Using AFM

The measurement method was slightly modified from our previous publication (Iwashita et al., 2014) to optimize for fixed samples. The measurements were carried out using AFM (Bioscope Resolve, NanoScope 9.4, Bruker), which was mounted on an inverted microscope (Nikon, ECLIPSE Ti2). A tipless silicon cantilever with a 20-μm borosilicate bead (Novascan) was used. The spring constant of the cantilever was calibrated using the thermal noise method in air. We chose cantilevers with the same spring constant (nominal value: 0.03 N/m; actual value: 0.07 N/m) and used them for acute and fixed slices individually to avoid cross-contamination of the remaining fixative in acute condition. The applied force was 10 nN. The measurement was done under physiological conditions for the acute slices (37°C) and at room temperature (25°C) for fixed slices. The force curves were acquired using the contact mode. Bright field images were acquired by a CMOS camera (Hamamatsu, ORCA-Flash4.0, C13440-20CU) to determine the measured region. The obtained force curves were analyzed to calculate the stiffness fit with the Hertzian model (spherical) using NanoScope Analysis 1.9 software (Bruker). Supplementary Table 3 describes the parameters for measurement.

### Immunostaining

To confirm the measured regions, the acute slices were immediately fixed with 4% PFA for 1 h at room temperature after measurement for DAPI staining. For immunohistochemistry, the adjacent cryosections of stiffness measured slices were incubated in 0.5% Triton-X 100/PBS for permeabilization for 10 min and then a 2% BSA/0.1% Triton-X-100 solution for 2 h for blocking followed by washing with PBS. Subsequently, the cryosections were incubated with primary antibodies for overnight at 20°C and then incubated with secondary antibodies for 2 h at room temperature followed by washing with PBS. The primary and secondary antibodies used in this study were rabbit anti-Tbr1 (1:500; Abcam, ab31490), rat anti-Ctip2 (1:500; Abcam, ab18465), and mouse anti-Satb2 (1:100; Abcam, ab51502). The secondary antibodies were Alexa 488-, 555-, and 647-conjugated (1:500; Molecular probes). DAPI was used to counter stain nuclei. Stained samples were mounted with PermaFluro (Thermo Fischer Scientific) and then observed using an upright confocal laser microscopy (Nikon, A1R-MP) and Panoramic Scan II (3DHISTECH).

### Statistical Analysis

All statistical analyses were performed using Prism 8 (GraphPad). A two-tailed unpaired *t*-test was applied to compare two conditions, and one-way analysis of variance (ANOVA) and the Tukey *post hoc* test were applied to compare more than three conditions. Differences were considered significant at ^∗^*P* < 0.05, ^∗∗^*P* < 0.01, ^∗∗∗^*P* < 0.001, and ^****^P < 0.0001. Error bars in graphs are represented as the mean ± SEM.

## Results

### Glyoxal Fixative Maintained Brain Structures as Well as PFA

**FIGURE 1:**
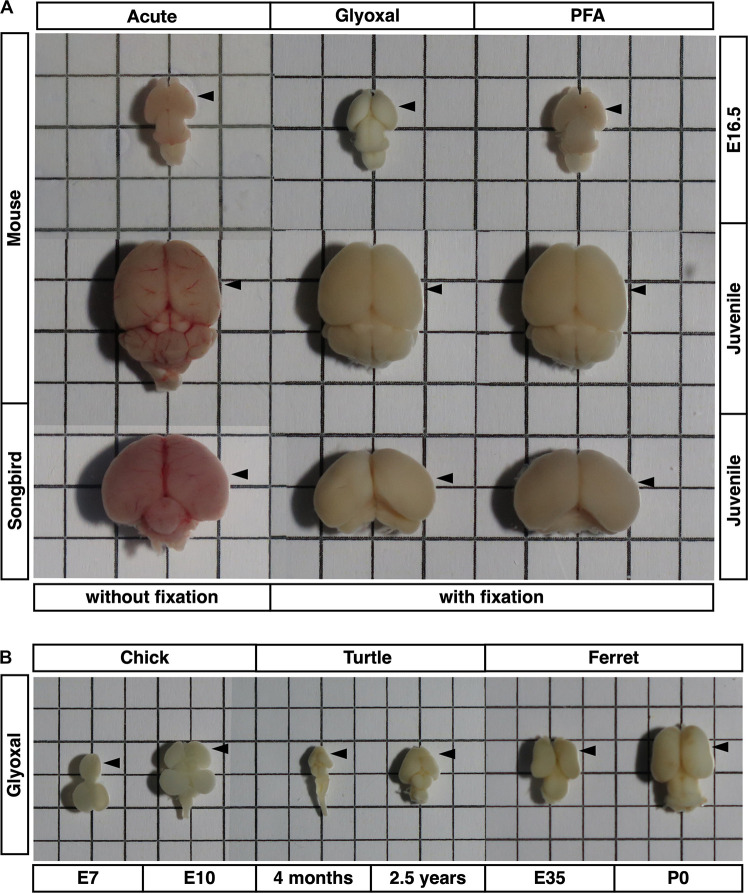
Brains used in this study. Brain sizes and morphologies of five animals (mouse, songbird, chick, turtle, and ferret). **(A)** Embryonic mouse brains at E16.5, 4 weeks mouse brains, and 30–40 dph songbird brains used in Figures 2–5. Fixation methods (PFA or glyoxal) are indicated. Acute: brains without fixation. **(B)** Chick brains at E7 and E10, turtle brains at 4 months and 2.5 years old, and ferret brains at E35 and P0 used in Figure 6. All brains were fixed with glyoxal. The grid has a resolution of 5 mm. Arrow heads: positions of stiffness measured in slices.

**FIGURE 2:**
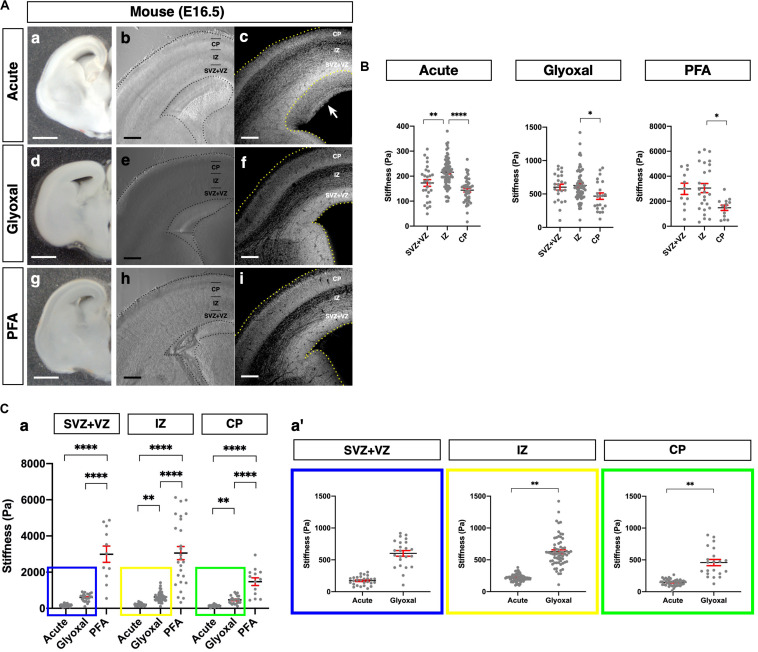
Comparison of tissue stiffness in different fixative conditions. **(A)** (a, d, and g) Representative images of brain slices: (a) acute, (d) glyoxal-fixed, and (g) PFA-fixed. (b, e, and h) Phase-contrast images of brain slices set on AFM: (b) acute, (e) glyoxal-fixed, and (h) PFA-fixed. (c, f, and i) DAPI images after measurement. Acute slice was fixed with PFA: (c) acute, (f) glyoxal-fixed, and (i) PFA-fixed. **(B)** Cortical stiffness measured by AFM. **(C)** (a) Comparison of stiffness in 3 measurement regions (SVZ+VZ, IZ, and CP). (a′) Magnified views of insets in (a). Each color corresponds to the measurement region. Blue, SVZ+VZ; yellow, IZ; green, CP. SVZ, subventricular zone; VZ, ventricular zone; IZ, intermediate zone; CP, cortical plate; scale bar: 1 mm for **(A)** (a, d, and g); 200 μm for **(B)** (b, c, e, f, h, and i). An arrow indicates an expanded proliferative region during measurement. One-way ANOVA with Tukey post hoc test; *P* < 0.05 (*), *P* < 0.01 (**), *P* < 0.001 (***), and *P* < 0.0001 (****) for **(B)** and **(C)**. Error bars in graphs are represented as the mean ± SEM.

**FIGURE 3:**
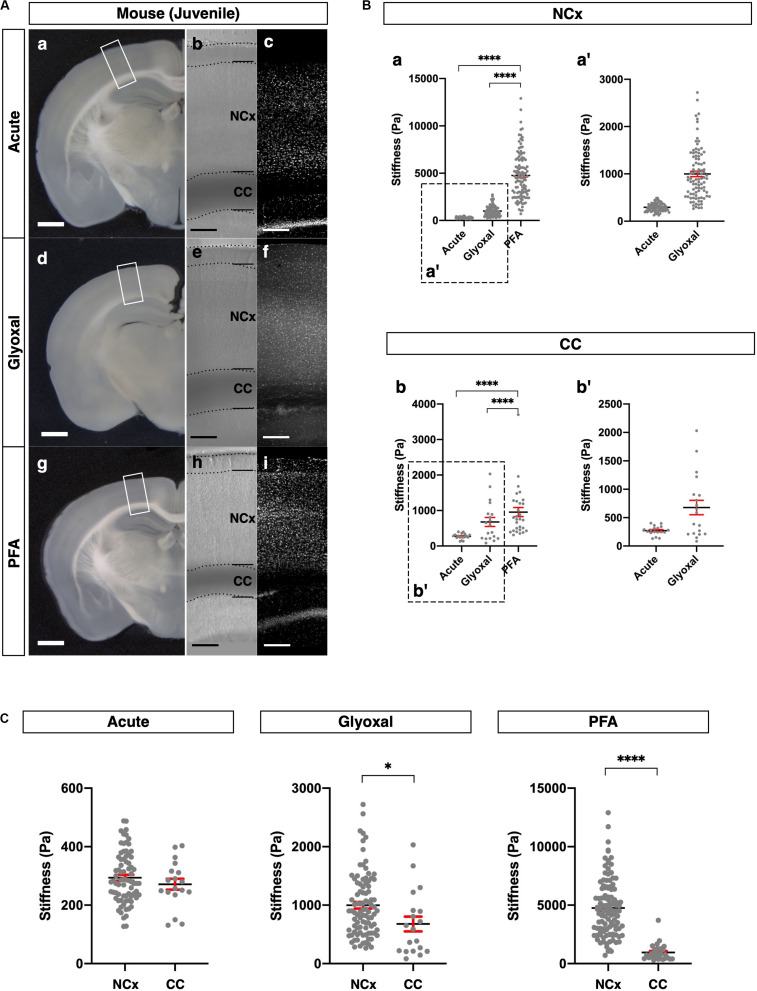
Comparison of tissue stiffness in juvenile mouse brains in different fixatives. **(A)** (a, d, and g) Representative images of 4-week-old mouse brain slices: (a) acute, (d) glyoxal-fixed, and (g) PFA-fixed. (b, e, and h) Phase-contrast images of brain slices set on AFM: (b) acute, (e) glyoxal-fixed, and (h) PFA-fixed. Note that CC shows dark color because of an optical filter setting. (c, f, and i) DAPI images: (c) acute, (f) glyoxal-fixed, and (i) PFA-fixed. **(B)** Comparison of stiffness in NCx (a) and CC (b) in different fixatives. (a′ and b′) Magnified views of insets in (a and b), respectively. **(C)** Comparison of stiffness between NCx and CC in each fixative. NCx, neocortex; CC, corpus callosum; scale bar: 1 mm for **(A)** (a, d, and g); 200 μm for **(A)** (b, c, e, f, h, and i). One-way ANOVA with Tukey post hoc test; *P* < 0.0001 (****) for **(B)**. Two-tailed unpaired *t*-test; *P* < 0.05 (*) and *P* < 0.0001 (****) for **(C)**. Error bars in graphs are represented as the mean ± SEM.

**FIGURE 4:**
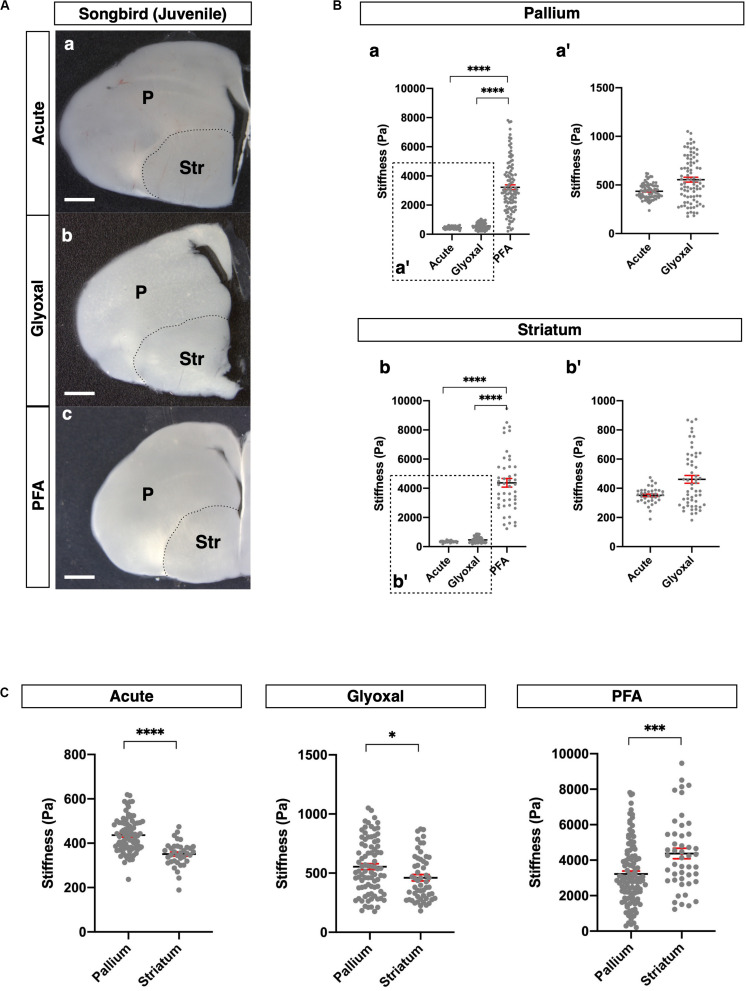
Comparison of tissue stiffness in juvenile songbird brains in different fixatives. **(A)** Representative images of juvenile songbird brain slices: (a) acute, (b) glyoxal-fixed, and (c) PFA-fixed. **(B)** Comparison of stiffness in P (a) and Str (b) in different fixatives. (a′ and b′) Magnified views of insets in (a and b), respectively. **(C)** Comparison of stiffness between P and Str in each fixative. P, pallium; Str, striatum; scale bar: 1 mm. One-way ANOVA with Tukey post hoc test; *P* < 0.0001 (****) for **(B)**. Two-tailed unpaired *t*-test; *P* < 0.05 (*), *P* < 0.001 (***), and *P* < 0.0001 (****) for**(C)**. Error bars in graphs are represented as the mean ± SEM.

To obtain reliable stiffness values in post-fixed brains, the tissue structure itself, including the macroscopic architecture and microenvironment, must be maintained like living conditions. Therefore, we investigated effective fixative solutions to maintain brain structure *in situ*. For this purpose, we chose 4% PFA and 3% glyoxal solutions as fixatives. PFA is common in histological studies, and glyoxal is a small dialdehyde molecule that is reported to provide better morphological preservation and strong fixation of both proteins and RNAs at cellular resolution because of its rapid penetration (Bussolati et al., 2017; Richter et al., 2018). We tested the immersion fixation for embryonic brains, transcardial perfusion for juvenile brains, and as a control, acutely prepared brains without fixation. The sizes of the fixed brains were slightly smaller than the acute brains because of the shrinkage following fixation in both methods. There was no difference at macroscopic resolution between either fixative except the color of the fixed brains (Figure 1). The brains fixed with glyoxal exhibited a white color, while the PFA-fixed brains exhibited a pale pink color. The acutely prepared juvenile brains showed a red color because of blood cells. Sectioned glyoxal-fixed brains also exhibited a white color, low contrast, and low transparency (Figures 2A, 3A, 4A, 6). The nuclei stained by DAPI showed similar brain cytoarchitectures in both fixative solutions (Figures 2A, 3A). These results show that glyoxal has an ability equivalent to PFA to preserve brain structures using both immersion and perfusion-fixation methods.

### Glyoxal-Fixed Brains Remained Much Softer Than PFA-Fixed Ones and Maintained the Relative Stiffness Profile of Living Tissue

Next, we examined differences in brain tissue stiffness between PFA and glyoxal fixation in comparison to acutely prepared living brains using AFM. We initially measured the stiffness of the mouse embryonic brains at E16.5. We prepared coronal slices from PFA- and glyoxal-fixed brains and, as a reference, acutely from living brains. The dorsal cortices were divided into three regions: the CP, intermediate zone (IZ), and proliferative region, including SVZ and VZ (SVZ+VZ), based on the phase-contrast images (Figure 2A). Subsequently, the stiffness in each region was measured using AFM (Figure 2B). Consistent with our previous results using a different AFM system (Iwashita et al., 2014), the stiffness in the IZ showed significantly higher values than other regions in acutely prepared living slices. In both PFA- and glyoxal-fixed brains, the stiffness in the IZ was significantly higher than in the CP but like that in the SVZ+VZ. Notably, although overall stiffness values increased with fixation, the glyoxal-fixed brains showed much lower stiffness than PFA-fixed brains (Figure 2C).

We further compared the stiffness tendencies of PFA- and glyoxal-fixed brains and acutely prepared living brains using 4-week-old juvenile mice. We made coronal slices, including the hippocampus, from each condition (Figure 3A) and measured the stiffness in the NCx and corpus callosum (CC) (Figure 3B). Like the embryonic brains, the PFA-fixed slices showed the highest stiffness in both the NCx and CC, while the glyoxal-fixed slices showed much lower stiffness in both regions. We also found that the stiffness in the NCx was relatively higher than in the CC in acute slices (294 ± 10 and 271 ± 19 Pa, respectively), although the statistical significance was not identified (Figure 3C). The relative difference in stiffness between the NCx and CC became strikingly higher in PFA (4761 ± 232 and 956 ± 129 Pa, respectively) but only moderate in the glyoxal-fixed condition (998 ± 56 and 678 ± 127 Pa, respectively). Our results from embryonic and juvenile mouse brains indicate that fixation by glyoxal can fairly maintain the relative stiffness profile of the NCx, keeping the overall softness of brain tissue adequately compared to PFA fixation.

### Stiffness in Glyoxal-Fixed Brains Had Tendencies Like Living Brains in Songbirds

Subsequently, we investigated the effect of fixative solutions on tissue stiffness using other species. For this purpose, we measured stiffness in juvenile songbird brains (30–40 dph). Juvenile songbirds can begin to feed themselves around this age, and this developmental period is considered relevant to the weaning stage of mice around 4 weeks after birth. We prepared coronal slices containing pallium in the dorsal part and striatum (Str) in the ventral part of the brains and measured the stiffness in both regions (Figure 4A). As with the mouse brains, the stiffness in the PFA-fixed brain slices was dramatically higher than in the other conditions, while glyoxal-fixed brain slices did not show any significant differences from the acute slice (Figure 4B). Notably, the pallium was significantly stiffer than the Str in both the acute and glyoxal-fixed slices (Figure 4C). However, the Str was stiffer than the pallium in PFA-fixed brains (Figure 4C and Table 1B). Furthermore, the range of stiffness values per measured point tended to be extremely broad in many PFA-fixed brains. Altogether, these results imply that fixation by PFA is not suitable for obtaining consistent tissue-stiffness profiles. With the results of the mouse and songbird brains, we conclude that glyoxal fixation might provide stiffness that relatively fits living tissue.

**TABLE 1:** Summary of stiffness.

**(A) Stiffness in mouse** (Figures 2,3)							
	**E16.5**					**Juvenile**	
	**SVZ+VZ**	**IZ**	**CP**			**CC**	**NCx**
Acute	173 ± 13 Pa (27 points)	216 ± 7 Pa (71 points)	144 ± 8 Pa (42 points)			271 ± 19 (18 points)	294 ± 10 (76 points)
Glyoxal	601 ± 44 Pa (23 points)	628 ± 30 Pa (64 points)	468 ± 48 Pa (22 points)			678 ± 127 (19 points)	998 ± 56 (93 points)
PFA	2993 ± 448 Pa (11 points)	3054 ± 361 Pa (26 points)	1475 ± 212 Pa (13 points)			956 ± 129 (29 points)	4761 ± 232 (111 points)

E16.5: Four acute brains, four glyoxal-fixed brains, and two PFA-fixed brains. Juvenile: Three acute brains, four glyoxal-fixed brains, and three PFA-fixed brains.							
**(B) Stiffness in songbird** (Figure 4)							
			**Pallium**				**Striatum**

Acute			436 ± 8 Pa (83 points)				351 ± 10 Pa (36 points)
Glyoxal			554 ± 25 Pa (85 points)				461 ± 26 Pa (54 points)
PFA			3224 ± 165 Pa (114 points)				4373 ± 298 Pa (47 points)

Three brains for each condition.							
**(C) Stiffness in mouse and songbird categorized by expression of neuronal markers (Figure 5)**							
		**Mouse (Juvenile)**				**Songbird (Juvenile)**	
		**Layer VI**	**Layer V**	**Layers II-IV**		**HP**	**M**
Glyoxal		879 ± 105 Pa (20 points)	972 ± 90 Pa (22 points)	1167 ± 85 Pa (49 points)		632 ± 38 Pa (45 points)	466 ± 26 PA (40 points)

Four mouse brains and three songbird brains.							
**(D) Stiffness in chick, turtle, and ferret fixed with glyoxal (Figure 6)**							
**(a) Chick**							
**E7**					**E10**		
**Area 1**	**Area 2**	**Area 3**			**Area 1**	**Area 2**	**Area 3**

630 ± 44 Pa (60 points)	614 ± 41 Pa (67 points)	609 ± 47 Pa (51 points)			568 ± 41 Pa (30 points)	612 ± 43 Pa (46 points)	771 ± 66 Pa (25 points)

(a) Two brains (five slices) at E7 and one brain (two slices) at E10.							
**(b) Turtle**							
	**Area 1**		**Area 2**		**Area 3**		**Area 4**

4 months	612 ± 45 Pa (23 points)		641 ± 32 Pa (23 points)		543 ± 36 Pa (22 points)		836 ± 97 Pa (22 points)
2.5 years	571 ± 30 Pa (18 points)		590 ± 19 Pa (28 points)		706 ± 42 Pa (17 points)		730 ± 58 Pa (17 points)

(b) One brain (two slices) at 4 months and one brain (two slices) at 2.5 years.							
**(c) Ferret**							
	**Area 1**	**Area 2**	**Area 3**	**Area 4**	**Area 5**	**Area 6**	**Area 7**

E35	396 ± 32 Pa (34 points)	472 ± 37 Pa (34 points)	455 ± 23 Pa (63 points)	402 ± 26 Pa (31 points)	487 ± 46 Pa (41 points)	610 ± 72 Pa (32 points)	578 ± 78 Pa (15 points)
P0	721 ± 58 Pa (16 points)	841 ± 69 Pa (18 points)	423 ± 19 Pa (44 points)	538 ± 49 Pa (22 points)	768 ± 51 Pa (23 points)	743 ± 85 Pa (19 points)	943 ± 136 Pa (13 points)
(c) One brain (four slices) at E35 and one brain (three slice) at P0.							

### Distinct Neuronal Subtypes in the Mouse and Songbird Pallia Exhibited Different Stiffness Profiles

The mammalian cerebral cortex consists of six layers, whereas the avian telencephalon consists of neuronal nuclei. Despite this difference in brain structure, mammalian and avian brains share representative neuronal markers, such as Satb2, Ctip2, and Tbr1 (Britanova et al., 2008; Nomura et al., 2013; Briscoe et al., 2018; García-Moreno et al., 2018; Tosches et al., 2018), although the conserved and diversified characteristics of these marker-positive neurons have not been fully addressed. To confirm whether glyoxal-fixed brains are eligible for comparatively analyzing the tissue stiffness of the pallium in distinct neuronal subtypes of different species, we compared the stiffness of tissue composed of cells expressing different neuronal markers in the mouse NCx and songbird pallium.

**FIGURE 5:**
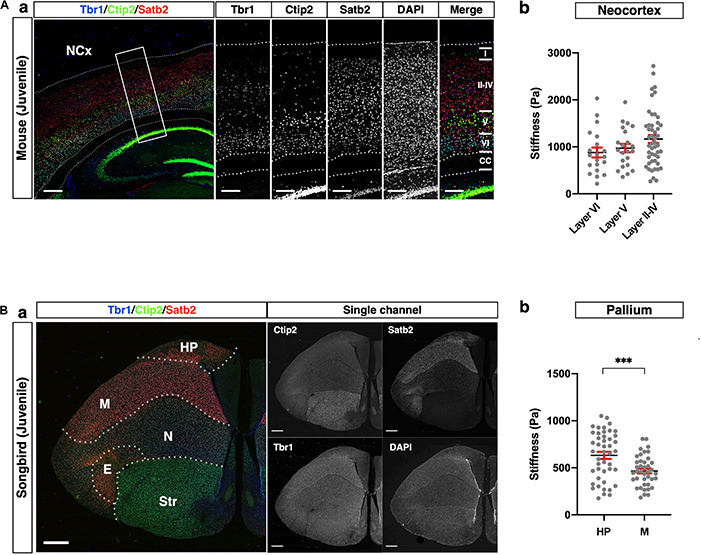
Comparison of stiffness in homologous regions between mouse and songbird. **(A)** (a) Expression of Satb2, Ctip2, and Tbr1 in juvenile mouse NCx. Satb2 was expressed broadly but relatively strongly in layers II–IV. A strong Ctip2 signal was observed in layer V. Tbr1 was expressed in layer VI. (b) Stiffness in each layer. **(B)** (a) Expression of Satb2, Ctip2, and Tbr1 in juvenile songbird brain. A strong Satb2 signal was observed in HP and M, while the Ctip2 signal was observed in the entire brain. Tbr1 was expressed in the M and N. (b) Regional stiffness in the pallium. NCx, neocortex; HP, hyperpallium; M, mesopallium; E, entopallium; Str, striatum; scale bar: 300 μm for **(A)**; 1 mm for **(B)**. Two-tailed unpaired *t*-test; *P* < 0.001 (***) for **(B)**. Error bars in graphs are represented as the mean ± SEM.

As reported previously (Molyneaux et al., 2007; Srinivasan et al., 2012; Hanashima and Toma, 2015), cortical layers in mice were divided as follows: Satb2 single-positive neurons in the upper layer (layers II–IV), Ctip2 strongly positive neurons in the middle part of the NCx (layer V), and Tbr1-positive neurons in the deeper layer (layer VI) (Figure 5A). The stiffness in layers II–IV was relatively higher than the other layers, but no significant differences existed between the layers. Interestingly, the stiffness in the NCx was based on the criterion of marker expression showing a monotonical increase along the apico-basal axis, that is, from the ventricular to the pial surface (Figure 5A).

The pallium of songbirds consists of the hyperpallium (HP), mesopallium (M), nidopallium (N), and entopallium (E) (Figure 5B; Reiner, 2005; Medina and Abellán, 2009). The songbird pallium shares neuronal markers with the mouse brain. Satb2 was strongly expressed in the HP and M, while Tbr1 was strongly expressed in the M and N. Ctip2 was expressed in the entire brain, including the ventral part (Str), while it was relatively weaker in the HP. We compared the regional stiffness in the pallium based on the expression level of the neuronal marker and found that the HP was stiffer than the M region (Figure 5B). These results indicate that the neuronal populations distinguished by subtype-specific markers exhibited different mechanical properties, particularly stiffness, in the mouse NCx and songbird pallium.

### Pallium in Amniotes Exhibited Species-Specific Stiffness Profiles

**FIGURE 6:**
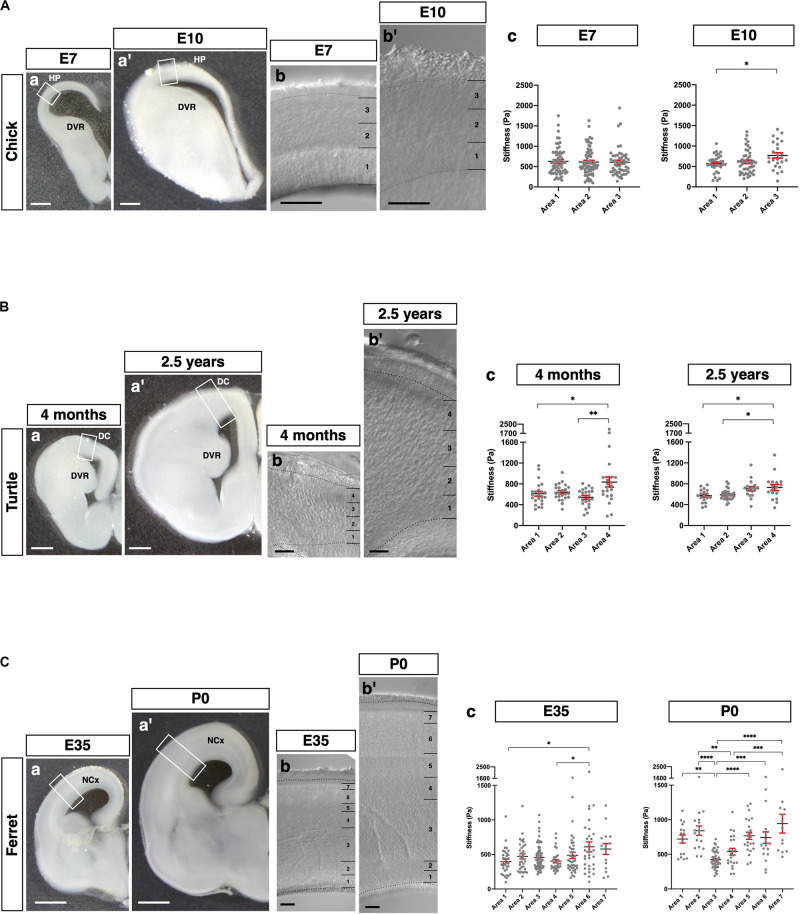
Species-specific stiffness profiles in amniote brains. **(A)** (a and a′) Representative images of the chick brains (E7 and E10, respectively) fixed with glyoxal. (b and b′) Phase-contrast image of the HP (E7 and E10, respectively), which was divided into three areas. (c) Stiffness in the HP along the apico-basal axis. **(B)** (a and a′) Representative images of the turtle brains (4 months and 2.5 years, respectively) fixed with glyoxal. (b and b′) Phase-contrast image of the DC (4 months and 2.5 years, respectively), which was divided into four areas. (c) Stiffness in the DC along the apico-basal axis. **(C)** (a and a′) Representative images of the ferret brains (E35 and P0, respectively) fixed with glyoxal. (b and b′) Phase-contrast image of the NCx (E35 and P0, respectively). The NCx was divided into seven areas. (c) Stiffness in the NCx along the apico-basal axis. HP, hyperpallium; DVR, dorsal ventricular ridge; DC, dorsal cortex; NCx, neocortex; scale bar: 500 μm for **(A)** (a and a′) and B (a and a′); 1 mm for **(C)** (a and a′); 200 μm for **(A)** (b and b′), **(B)** (b and b′), and **(C)** (b and b′). One-way ANOVA with Tukey post hoc test; *P* < 0.05 (*), *P* < 0.01 (**), *P* < 0.001 (***), and *P* < 0.0001 (****) for **(A–C)**. Error bars in graphs are represented as the mean ± SEM.

Finally, we applied our glyoxal brain-fixation method to comparatively analyze the stiffness of various species using chicks (E7 and E10), turtles (4 months and 2.5 years), and ferrets (E35 and P0). These brains were fixed by glyoxal immediately after dissection and shipped overseas. Subsequently, slices were made to measure their stiffness in one place using the same conditions and an AFM system. We prepared coronal brain slices of each animal and then measured the stiffness in the pallium along the apico-basal axis (Figure 6). The embryonic chick HP was divided into three areas (1, 2, and 3) corresponding to the VZ and the lower and upper neuronal zones based on the phase-contrast image (Figure 6A). We found a gradual increase of stiffness along the apico-basal axis at E10, the softest region being the VZ, which is occupied by neural progenitor cells (Nomura et al., 2016). Compared to E10, no stiffness gradient was observed at E7. The turtle dorsal cortex (DC) was divided into four areas corresponding to the VZ and cortical layers III, II, and I (Figure 6B). The major cell type in the turtle DC is neurons at these stages, especially Satb2-positive and Ctip2-positive neurons, which are intermingled in layers II and III (Areas 2 and 3 in Figure 6B; Nomura et al., 2013, 2018; Suzuki and Hirata, 2014; Tosches and Laurent, 2019). We found significantly higher stiffness in Area 4 at 4 months. However, the stiffness of Areas 1 and 3 had no significant difference. The 2.5 years turtle DC showed gradually increasing stiffness along the apico-basal axis, the softest region at the VZ, the most apical region. We also examined the stiffness of the ferret NCx, which was divided into seven areas along the apico-basal axis at E35 and P0 (Figure 6C). The NCx stiffness exhibited a much different profile in ferrets, showing a higher stiffness in Areas 6 and 7, a region corresponding to the CP, and a flattened pattern of stiffness from Areas 1–5 at E35. Intriguingly, a parabolic stiffness pattern with the lowest valley in Area 3, a region corresponding to the outer SVZ (OSVZ) (Reillo et al., 2011; Reillo and Borrell, 2012), was observed at P0. In embryonic mouse brains, the IZ was stiffer than other regions (Figure 2), but the IZ in P0 ferret brain (Areas 4–5) was softer, while the VZ and the inner SVZ (ISVZ) (Areas 1 and 2, respectively) and the CP (Areas 6 and 7) were stiffer. These differences in mechanical properties during development might provide distinct physical cues that contribute to the species-specific morphologies of the respective animals’ mature brains (see section “Discussion”).

## Discussion

The highlighted findings of this study are as follows: (1) Glyoxal-fixed brains can fairly maintain the relative stiffness of living tissue, and (2) brains fixed with glyoxal exhibit species-specific stiffness profiles.

Regarding (1), glyoxal has several advantages in fixation. It was reported several decades ago as an alternative fixative to formalin (Wicks and Suntzeff, 1943). Its simple dialdehyde structure enables it to penetrate cells rapidly and preserve the immunoreactivity of proteins (Richter et al., 2018). Its preservation of nucleic acid is also of acceptable quality for fluorescent *in situ* hybridization and next-generation sequencing analysis (Bussolati et al., 2017). Importantly, glyoxal is easily handled due to its low toxicity—that is, its lack of evaporation from solution. We tested the standard fixation methods, that is, immersion fixation and transcardial perfusion. Glyoxal-fixed brains turned white during fixation in both methods (Figure 1), a color that was maintained in the sectioned brains and resulted in low contrast and transparency compared to the acute and PFA-fixed brain slices (Figures 2–4, 6). This effect sometimes creates difficulty distinguishing tissue structures under a microscope. The glyoxal-fixed brains also became brittle, so trimming the region of interest in advance when using tissue with uniform structure and large, thick sections might be necessary.

The stiffness in the fixed tissue was higher than that of living tissue irrespective of brain size and fixation method, that is, PFA or glyoxal fixation. Remarkably, however, brain tissue fixed with glyoxal remained much softer than PFA and maintained relative stiffness like living conditions in most cases (Figures 2–4). Since glyoxal is a small molecule, non-cross-linked molecules might be washed out by PBS replacement. In contrast, the stiffness in the PFA-fixed brains was approximately 10 times higher than in living brains. Interestingly, the stiffness in the juvenile mouse CC was not so high, even in the PFA-fixed brain (Figure 3). The previously reported stiffness of white matter in rat cerebellums was 294 ± 74 Pa in living tissue (Christ et al., 2010). The stiffness of the CC measured in this study was 271 ± 19 Pa in acute slices, which fits the range found by a previous report (Christ et al., 2010), while it was 678 ± 127 Pa in the glyoxal solution and 956 ± 129 Pa in the PFA solution. The NCx, however, showed much larger differences in stiffness, with 294 ± 10 Pa in the acute, 998 ± 56 Pa in the glyoxal, and 4761 ± 232 in the PFA solutions (Table 1A). It is unclear why the CC showed lower stiffness in the fixed brains. Axon bundles in the CC are tightly wrapped with myelin sheaths consisting of lipids, so the lipid-rich myelin structure might affect the cross-linking of fixatives, lowering overall stiffness.

Regarding (2), we examined the stiffness profiles of homologous structures across species (Figure 5 and Table 1C) and stage-dependent stiffness profiles in the extant amniotes (Figure 6 and Table 1D).

First, we compared the stiffness of the homologous structure shared by the mouse NCx and songbird HP to investigate whether the pallial cytoarchitecture affected the tissue stiffness (Figure 5). In the mammalian NCx, each cortical layer consists of specific neural subtypes distinguished by layer-specific transcription factors, such as Ctip2, Satb2, and Tbr1 (Molyneaux et al., 2007; Britanova et al., 2008; Srinivasan et al., 2012; Hanashima and Toma, 2015). In contrast, the songbird HP, a homologue of the mammalian NCx, does not exhibit layer structures. Since the expression of orthologous genes does not discriminate between structural differences, we did not divide further areas in the songbird HP. Instead, neural subtype-specific genes allowed us to distinguish each pallial compartment in the songbird brain (Figure 5B). However, we could not find conserved stiffness patterns in neuronal populations expressing specific neuron-subtype markers; rather, neuronal subtypes in distinct pallial regions exhibited species-specific stiffness. This result is consistent with our previous report (Nomura et al., 2018): the expression of cell type-specific transcription factors does not confer evolutionarily conserved cellular characteristics, which disputes the theory of cell-type homology based on the expression of orthologous gene expression. Nevertheless, integrating the histological and functional analyses of neurons may be necessary for direct interspecies stiffness comparisons in the future.

Second, we applied glyoxal fixation to several amniotes to investigate the stiffness of their pallia exhibiting different morphologies. We chose representative species based on phylogenetic and histological reasons (Puelles et al., 2000, 2016, 2019; de Juan Romero and Borrell, 2015; Medina et al., 2017, 2019; Desfilis et al., 2018; Pessoa et al., 2019; García-Moreno and Molnár, 2020). We successfully obtained species-specific stiffness profiles using chick, songbird, turtle, mouse, and ferret brains, which are classified as birds, reptiles, and mammals. Although birds and turtles are Sauropsids, the structure of their pallium exhibits a different cytoarchitecture. The DC in the reptile pallium has a layered structure (Connors and Kriegstein, 1986; Crockett et al., 2015; Tosches et al., 2018), whereas the HP in the bird pallium consists of neuronal nuclei (Reiner, 2005; Medina and Abellán, 2009). Mice and ferrets have an enlarged NCx with distinct complexities, as the mouse NCx is lissencephalic, while the ferret’s exhibits remarkable gyrification (de Juan Romero et al., 2015), although six-layered cortical lamination is extensively shared by both species.

We found stage-dependent and species-specific stiffness in pallia among amniotes. For instance, a monotonous pattern of stiffness was observed in chicks at E7 (Figure 6A). This tendency corresponds to our previous report (Iwashita et al., 2014), showing that no significant differences among cortical layers were detected in early neurogenesis in mice (E12.5–14.5). In contrast, the embryonic ferret NCx showed a different tendency, indicating a monotonous pattern in the proliferative region, including the VZ, ISVZ, OS, IZ, and SP but with a relatively higher stiffness in the CP (Figure 6C). Notably, the ferret NCx at P0 exhibited a catenary stiffness pattern. In contrast to embryonic mouse brains, the CP, ISVZ, and VZ showed higher stiffness values than other layers, while the IZ showed lower stiffness. The OSVZ, a distinctive layer in species with a folded NCx, including ferrets, monkeys, and humans (Smart, 2002; Fietz et al., 2010; Reillo et al., 2011), showed the lowest stiffness. These differences in mechanical properties might affect cellular behavior during brain development. The extracellular matrix (ECM) determines the stiffness of tissues; in fact, recent studies have identified tissue-specific ECMs and their effects on cellular behavior, including proliferation, fate determination, and migration (Fietz et al., 2012; Long and Huttner, 2019; Ueno et al., 2019). Distinct stiffness might control cellular behavior during development and contribute to the different morphologies of brains, such as lissencephalic surfaces in mice, gyrencephalic surfaces in ferrets, and neuronal slabs in birds.

In comparing the matured pallia, gradually increasing stiffness along the apico-basal axis was observed in the 2.5 years turtle DC, which was derived from the dorsal pallium, a homologous region of the mammalian NCx (Figure 6B). This gradient pattern was also observed in the mouse juvenile NCx (Figure 5A). The outer layer of the NCx in mice is terminated with apical dendrites, while the DC in turtles is occupied by densely packed dendrites (Connors and Kriegstein, 1986; Crockett et al., 2015). Cytoskeleton-rich processes might contribute to determining regional stiffness. Further systematic studies using different stages and animals are required to confirm that this stiffness gradient is common in pallia with laminar structures.

## Conclusion

**FIGURE 7:**
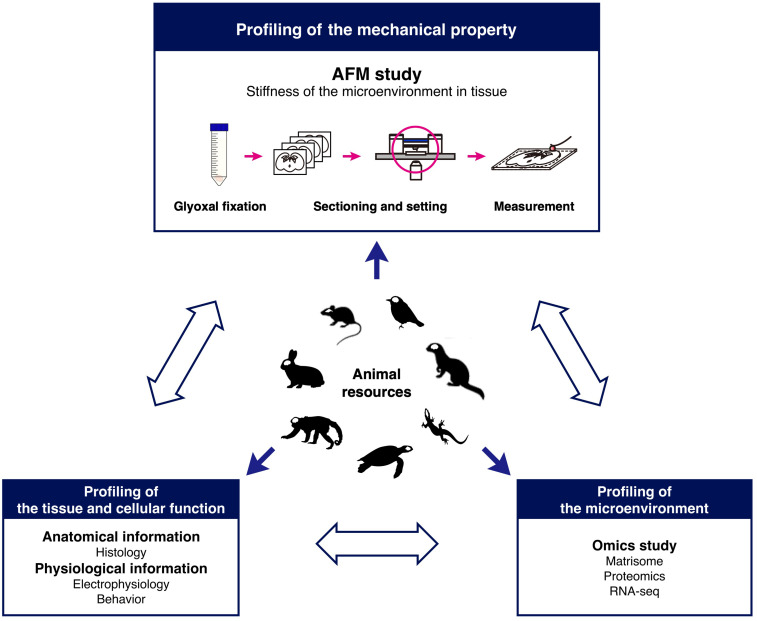
Research model for comparative analysis of tissue mechanics. The schematic shows a model to investigate tissue stiffness in animals to understand the role of the microenvironment in development.

In conclusion, glyoxal fixation can be applicable to the study of the mechanical properties of the brain in combination with AFM. Our findings based on our method strongly suggest that species-specific microenvironments might exist in the brain and that distinct mechanical properties could provide different cues to neural cells to form diverse brain structures as a result of migration during development. To identify the interactions between cells and microenvironments, further systematic analysis is required. Therefore, we propose a novel research model for brain development based on the mechanical properties of microenvironments (Figure 7). Combining stiffness data with histological and omics (Naba et al., 2016) analysis enables systematically and quantitatively analyzing correlations between mechanical properties and molecules in developing brains. This research model using glyoxal-fixed brains could help elucidate the diversity of brain structures.

## Data Availability Statement

The raw data supporting the conclusions of this article will be made available by the authors, without undue reservation.

## Ethics Statement

The animal study was reviewed and approved by the Committee of Korea Brain Research Institute.

## Author Contributions

MI and YK designed the research concept and wrote the manuscript. MI prepared the mouse brains, measured the stiffness, and analyzed the results. TN prepared the turtle and chick brains and wrote the manuscript. TS and FM prepared the ferret brains. SK prepared the songbird brains. All the authors read and approved the final manuscript.

## Conflict of Interest

The authors declare that the research was conducted in the absence of any commercial or financial relationships that could be construed as a potential conflict of interest.
