# Supplementary material for: Comparative Analysis of Brain Stiffness Among Amniotes Using Glyoxal Fixation and Atomic Force Microscopy
Source: Front Cell Dev Biol. 2020 Sep 11;8:574619. doi: 10.3389/fcell.2020.574619 (PMC7517470; doi:10.3389/fcell.2020.574619)
Supplement: Supplementary file 1 [file Data_Sheet_1.PDF]

Supplementary Material

Comparative analysis of brain stiffness among amniotes using glyoxal fixation and atomic force microscopy

Authors
Misato Iwashita[1*], Tadashi Nomura[2], Taeko Suetsugu[3], Fumio Matsuzaki[3], Satoshi Kojima[1], and Yoichi Kosodo[1*]

Affiliations
[1] Korea Brain Research Institute, 61, Cheomdan-ro, Dong-gu, Daegu, 41062, Republic of Korea
[2] Developmental Neurobiology, Kyoto Prefectural University of Medicine, INAMORI Memorial Building, 1-5 Shimogamo-Hangi cho, Sakyoku, Kyoto 606-0823, Japan
[3] RIKEN Center for Biosystems Dynamics Research, 2-2-3 Minatojima-minamimachi, Chuo-ku, Kobe, Hyogo 650-0046, Japan
*Correspondence: misato.iwashita@kbri.re.kr, kosodo@kbri.re.kr

Supplementary Table 1. Media components used for the measurement of the acute slice for juvenile mouse

Slicing ACSF for mouse

Components	Final conc. (mM)	Company	Catalog number
Sucrose	175	GENERAY BIOTECH	0335-500G
NaCl	20	Sigma-Aldrich	S9888-50G
KCl	3.5	Sigma-Aldrich	P9541-500G
NaH_2PO_4	1.25	Sigma-Aldrich	71505-1KG
$NaHCO_3$	26	Sigma-Aldrich	S5761-500G
$MgCl_2$	1.3	Sigma-Aldrich	68475-100ML-F
Glucose	11	JUNSEI	64220S0650

Measurement ACSF for mouse

Components	Final conc. (mM)	Company	Catalog number
NaCl	120	Sigma-Aldrich	S9888-50G
KCl	3.5	Sigma-Aldrich	P9541-500G
NaH_2PO_4	1.25	Sigma-Aldrich	71505-1KG
$NaHCO_3$	26	Sigma-Aldrich	S5761-500G
$MgCl_2$	1.3	Sigma-Aldrich	68475-100ML-F
Glucose	11	JUNSEI	64220S0650
$CaCl_2$	2	DUKSAN	3459

Supplementary Table 2. Media components used for the measurement of the acute slice for juvenile songbird

Slicing ACSF for songbird

Components	Final conc. (mM)	Company	Catalog number
Sucrose	119	GENERAY BIOTECH	0335-500G
KCl	2.5	Sigma-Aldrich	P9541-500G
NaH_2PO_4	1	Sigma-Aldrich	71505-1KG
$NaHCO_3$	26.2	Sigma-Aldrich	S5761-500G
$MgCl_2$	1.3	Sigma-Aldrich	68475-100ML-F
Glucose	11	JUNSEI	64220S0650
$CaCl_2$	2.5	DUKSAN	3459

Measurement ACSF for songbird

Components	Final conc. (mM)	Company	Catalog number
NaCl	119	Sigma-Aldrich	S9888-50G
KCl	2.5	Sigma-Aldrich	P9541-500G
NaH_2PO_4	1	Sigma-Aldrich	71505-1KG
$NaHCO_3$	26.2	Sigma-Aldrich	S5761-500G
$MgCl_2$	1.3	Sigma-Aldrich	68475-100ML-F
Glucose	11	JUNSEI	64220S0650
$CaCl_2$	2.5	DUKSAN	3459

Supplementary Table 3. Parameters used for AFM measurement

**Parameter**	
Ramp Size	10 µm
Ramp Rate	1.00 Hz
Forward Velocity	20.0 µm/s
Reverse Velocity	20.0 µm/s
Trigger Threshold	10 nN
Trigger Safety	10 nN
Spring constant (actual)	0.07 N/m
Bead Radius	10 µm
Sample Poisson's Ratio	0.5
Modulus Fitting Model	Hertzian (Spherical)
